# Examining Development Processes for Text Messaging Interventions to Prevent Cardiovascular Disease: Systematic Literature Review

**DOI:** 10.2196/12191

**Published:** 2019-03-29

**Authors:** Ignacio Ricci-Cabello, Kirsten Bobrow, Sheikh Mohammed Shariful Islam, Clara K Chow, Ralph Maddison, Robyn Whittaker, Andrew J Farmer

**Affiliations:** 1 Balearic Islands Health Research Institute Palma de Mallorca Spain; 2 Atención Primaria Mallorca, IB-Salut Palma de Mallorca Spain; 3 Ciber de Epidemiologia y Salud Publica Madrid Spain; 4 Chronic Disease Initiative for Africa Cape Town South Africa; 5 Division of Diabetic Medicine and Endocrinology Department of Medicine University of Cape Town Cape Town South Africa; 6 Radcliffe Observatory Quarter Nuffield Department of Primary Care Health Sciences University of Oxford Oxford United Kingdom; 7 Institute for Physical Activity and Nutrition Deakin University Geelong Australia; 8 Sydney Medical School Faculty of Medicine and Health University of Sydney Sydney Australia; 9 The George Institute for Global Health University of New South Wales Sydney Australia; 10 Westmead Applied Research Centre Faculty of Medicine and Health University of Sydney Sydney Australia; 11 Department of Cardiology Westmead Hospital Sydney Australia; 12 National Institute for Health Innovation The University of Auckland Auckland New Zealand; 13 Waitemata District Health Board Auckland New Zealand

**Keywords:** systematic review, cardiovascular disease, telemedicine, text messaging, methods

## Abstract

**Background:**

Interventions delivered by mobile phones have the potential to prevent cardiovascular disease (CVD) by supporting behavior change toward healthier lifestyles and treatment adherence. To allow replication and adaptation of these interventions across settings, it is important to fully understand how they have been developed. However, the development processes of these interventions have not previously been systematically examined.

**Objective:**

This study aimed to systematically describe and compare the development process of text messaging interventions identified in the Text2PreventCVD systematic review.

**Methods:**

We extracted data about the development process of the 9 interventions identified in the Text2PreventCVD systematic review. Data extraction, which was guided by frameworks for the development of complex interventions, considered the following development stages: intervention planning, design, development, and pretesting. Following data extraction, we invited the developers of the interventions to contribute to our study by reviewing the accuracy of the extracted data and providing additional data not reported in the available publications.

**Results:**

A comprehensive description of the development process was available for 5 interventions. Multiple methodologies were used for the development of each intervention. Intervention planning involved gathering information from stakeholder consultations, literature reviews, examination of relevant theory, and preliminary qualitative research. Intervention design involved the use of behavior change theories and behavior change techniques. Intervention development involved (1) generating message content based on clinical guidelines and expert opinions; (2) conducting literature reviews and primary qualitative research to inform decisions about message frequency, timing, and level of tailoring; and (3) gathering end-user feedback concerning message readability, intervention acceptability, and perceived utility. Intervention pretesting involved pilot studies with samples of 10 to 30 participants receiving messages for a period ranging from 1 to 4 weeks.

**Conclusions:**

The development process of the text messaging interventions examined was complex and comprehensive, involving multiple studies to guide decisions about the scope, content, and structure of the interventions. Additional research is needed to establish whether effective messaging systems can be adapted from work already done or whether this level of development is needed for application in other conditions and settings.

## Introduction

### Background

Worldwide, cardiovascular disease (CVD) is a major cause of premature morbidity and mortality [1]. Observational studies have shown that a few potentially modifiable risk factors account for most of the risk of CVD outcomes [2,3]. These risk factors, which include abnormal blood lipids, smoking, diabetes, and high blood pressure, are common and often occur together in the same individual. For example, more than two-thirds of people with diabetes also have high blood pressure [4]. There is good evidence now that low-cost medications and changes in lifestyle can improve risk profiles for individuals [5]. However, suboptimal adherence to prescribed medication and lifestyle modification is a major barrier to the health benefits of these evidence-based treatments [6].

Mobile communications technology has the potential to support behavior change and treatment adherence by facilitating remote, interactive, and timely access to relevant information and providing context-specific support and prompts to action [7]. The simplest and most widely available mobile communications standard is the short message service (SMS) text message. Results from some but not all trials suggest that SMS text message interventions may be a useful adjunct to usual care for the prevention and management of CVD [8-10]. The quality and success of these interventions partially depend on how they have been developed. Guidelines for the development of digital [11] and mobile Health (mHealth) behavior change interventions [12,13] now exist. They propose a number of steps to follow to maximize intervention benefit, including formative research for insights into the target audience and health behavior, designing the text messaging program, pretesting the text messaging program concept and messages, and revising the text messaging program.

However, the development process of mHealth interventions is frequently underreported and not well understood, having been described as “hidden within a black box” [14]. Understanding how mHealth interventions are developed is crucial to allow replication and adaptation of successful interventions across settings—a necessary step to accumulating evidence around the effectiveness of these interventions. In 2016, the *International Consortium of Text2PreventCVD Trial Collaborators Group* was established as part of an initiative to gather and analyze individual participant data from randomized clinical trials identified as part of a systematic literature review of text messaging interventions to prevent CVDs [15]. This international consortium offered a unique opportunity for an in-depth examination of the development processes of these interventions, based on information directly supplied by the developers of the interventions (rather than merely relying on data reported in available publications, as commonly done in conventional systematic literature reviews).

### Objective

The aim of this study was to systematically describe and compare the processes followed to develop the text messaging interventions for the prevention and management of CVD that were included in the Text2PreventCVD meta-analysis.

## Methods

### Design

We undertook a systematic description and comparison of the development processes of the interventions included in the Text2PreventCVD systematic review (the results from the individual participant data meta-analysis examining the effectiveness of the interventions in the prevention of CVD will be published elsewhere).

### Identification of Studies

The protocol including the methods for the identification of trials included in the Text2PreventCVD systematic review is available elsewhere [15]. In short, 4 electronic databases of published studies (MEDLINE, EMBASE, PsycINFO, and Cochrane Library) and international trial registries were searched to identify potentially relevant studies. The eligibility of the retrieved references was assessed by 2 independent reviewers based on the following inclusion criteria: (1) randomized clinical trials of mobile phone SMS or text message intervention with a control group receiving standard care (no messages or some form of control message), (2) follow-up period of at least 6 months, (3) with a minimum 70% of completed follow-up of patients, (4) focusing on CVD primary and secondary prevention in men and women aged 18 years and older, (5) evaluating interventions applying at least 2 behavioral techniques to support behavior change (as an indicator of a comprehensive CVD prevention program rather than a single focus, eg, medication adherence reminders), and (6) with a minimum total sample size of 30 participants (as sample size was perceived to be a surrogate marker of study quality).

The principal investigators of the 9 selected trials [8,9,16-34] meeting the inclusion criteria described above were invited by email to join the International Consortium of Text2PreventCVD Trial Collaborators Group. Reminders were sent after a week to nonresponders, followed by approaching other investigators by email, phone calls, and fax. Investigators were requested to share their data after obtaining a signed agreement. Authors of 5 of the trials (Tobacco, Exercise and Diet Messages [TEXT ME] [9,19,32], Islam et al [22,33], Heart Exercise And Remote Technologies [Heart] [24-26,30], Mobile Phone Text Messages to Support Treatment Adherence in Adults With High Blood Pressure [StAR[ [8,18], and Text message and Internet-based comprehensive cardiac rehabilitation intervention [Text4Heart] [21,29]) agreed to participate by providing data on the development of their interventions.

### Data Collection

Before data extraction, 3 researchers (IRC, AF, and KB) designed a data extraction form in an iterative process. The Medical Research Council framework for the development of complex interventions [35] and other available frameworks [11,12,36,37] were used to ensure the data extraction form covered all relevant areas in the development of mHealth interventions. The resulting data extraction form covered the following 4 domains: intervention planning, intervention design, intervention development, and intervention pretesting. The final form was reviewed and approved by the senior authors of the 5 trials included in this study. One researcher (IRC) then used the final form to extract all the relevant data available in the full texts and appendices of the manuscripts previously identified. Subsequently, the extracted information was sent by email to the authors of the 5 trials that agreed to take part in this study. They were requested to cross-check the accuracy of the extracted information and to provide additional details not reported in the available publications. When needed, we recontacted the authors for additional details or clarifications about the methods used to develop their intervention.

### Data Analysis

We carried out a narrative synthesis of the results, describing the different methods identified for developing the interventions. We classified the methods according to the 4 development stages considered (intervention planning, design, development, and pretesting). We calculated, in terms of frequency, the number of interventions that applied each of the methods identified and tabulated this information (Table 1). To allow head-to-head comparisons across interventions, we present in tables (Tables 2-4), and describe narratively, more detailed information about the methods used for the development of each intervention.

## Results

### Intervention Characteristics

We extracted information about the main characteristics of the selected mHealth interventions, including the level of customization of the messages, timing and frequency of delivering the messages, content (degree of personalization, readability, length, and presence of sender´s signature), directionality (unidirectional or bidirectional), and type of platform used to deliver the messages (see Multimedia Appendices 1 and 2). All the interventions were based on text messages containing less than 160 characters, which included the sender’s signature, and which were sent free of charge to participants. Most interventions included some degree of customization either in the content of the message (TEXT ME [9,19,32] and Heart [24-26,30]), preferred timing (Text4Heart [21,29] and StAR [8,18]), or language (StAR [8,18]). The number of messages sent per week ranged from 1 (StAR [8,18]) to 7 (Text4Heart [21,29]). Furthermore, 3 interventions (TEXT ME [9,19,32], Islam [22,33], and Heart [24-26,30]) used unidirectional messages, whereas 2 interventions (Text4Heart [21,29] and StAR [8,18]) used bidirectional messages (ie, allowing the participants to not only receive but also send messages).

Although the interventions generally aimed at preventing CVD, they varied in terms of their specific goal and population targeted. The TEXT ME intervention aimed at decreasing cardiovascular risk by encouraging lifestyle change in people with coronary heart disease in Australia [9,19,32]. The Text4Heart intervention aimed to support adherence to healthy lifestyle behaviors in adults with coronary heart disease in New Zealand [21,29]. The intervention developed by Islam et al [22,33] aimed to improve glycemic control in patients with type 2 diabetes in Bangladesh. The Heart intervention aimed to promote exercise capacity and physical activity behavior in people with ischemic heart disease in New Zealand [24-26,30]. The StAR intervention aimed to support treatment adherence and blood pressure control in adults with hypertension in South Africa [8,18].

### Intervention Development Process

All the interventions followed a comprehensive development process (or framework) that involved 1 or more studies to inform each development stage, including the planning, design, development, and pretesting of the interventions (Table 1).

#### Intervention Planning

A number of different information sources were used to identify the key behavioral issues, needs, and challenges the intervention was planned to address (Multimedia Appendix 3). These included stakeholder consultations (used in all interventions), literature reviews (TEXT ME [9,19,32], Text4Heart [21,29], Islam [22,33], and StAR [8,18]), examination of relevant theory (TEXT ME [9,19,32], Text4Heart [21,29], and Heart [24-26,30]), and preliminary qualitative research (StAR [8,18] and Heart [24-26,30]).

#### Intervention Design

Interventions were designed according to a wide range of behavior change theories (Table 2). Overall, 3 interventions (TEXT ME [9,19,32], Text4Heart [21,29], and Islam [22,33]) were based on multiple theories, whereas 2 interventions (Heart [24-26,30] and StAR [8,18]) relied on a single theory. All interventions were designed following multiple behavior change techniques (mean number of techniques per intervention=8, range=2 to 17). Most common techniques included general encouragement (used in 4 interventions), information about behavior-health link (3 interventions), information on consequences (3 interventions), time management advice (3 interventions), and goal setting (3 interventions).

**Table 1:** Frequency of the different methods used to inform the development process of the interventions identified.

Methods used to inform the development process of the interventions identified			Number of trials (trial name)
**Intervention planning**			
	**Information sources consulted to identify the key behavioral issues, needs, and challenges that the planned intervention was intended to address**		
		Consultation with experts, users, or other stakeholders	5 (TEXT ME^a^ [9,19,32], Text4Heart^b^ [21,29], Islam [22,33], Heart^c^ [24-26,30], and StAR^d^ [8,18])
		Literature reviews	4 (TEXT ME [9,19,32], Text4Heart [21,29], Islam [22,33], and StAR [8,18])
		Primary research with users	4 (TEXT ME [9,19,32], Text4Heart [21,29], Heart [24-26,30], and StAR [8,18])
		Examination of relevant theory	3 (TEXT ME [9,19,32], Text4Heart [21,29], and Heart [24-26,30])
**Intervention design**			
	**Theories used**		
		Social-cognitive theory [38]	2 (Text4Heart [21,29] and TEXT ME [9,19,32])
		Control theory [39]	1 (TEXT ME [9,19,32])
		Information-motivation-behavioral skills model [40]	1 (TEXT ME [9,19,32])
		Operant conditioning [41]	1 (TEXT ME [9,19,32])
		Theory of planned behavior [42]	1 (TEXT ME [9,19,32])
		Theory of reasoned action [43]	1 (TEXT ME [9,19,32])
		Common sense model [44]	1 (Text4Heart [21,29])
		Behavioral learning theory [45]	1 (Islam [22,33])
		Transtheoretical model of behavioral change [46]	1 (Islam [22,33])
		Self-efficacy theory framework [47]	1 (Heart [24-26,30])
		Integrated theory of behavior change [48]	1 (StAR [8,18])
		No theory used	0
	**Behavior change techniques used**		
		Provide general encouragement	4 (TEXT ME [9,19,32], Text4Heart [21,29], Heart [24-26,30], and StAR [8,18])
		Provide information about behavior-health link	3 (TEXT ME [9,19,32], Text4Heart [21,29], and StAR [8,18])
		Provide information on consequences	3 (TEXT ME [9,19,32], Text4Heart [21,29], and StAR [8,18])
		Prompt specific goal setting	3 (Text4Heart [21,29], Heart [24-26,30], and StAR [8,18])
		Time management	3 (TEXT ME [9,19,32], Text4Heart [21,29], and Heart [24-26,30])
		Prompt barrier identification	3 (TEXT ME [9,19,32], Text4Heart [21,29], and Heart [24-26,30])
		Set graded tasks	2 (TEXT ME [9,19,32] and Text4Heart [21,29])
		Provide instruction	2 (TEXT ME [9,19,32] and Text4Heart [21,29])
		Model or demonstrate the behavior	2 (Text4Heart [21,29] and Heart [24-26,30])
		Prompt self-monitoring of behavior	2 (Text4Heart [21,29] and TEXT ME [9,19,32])
		Provide information about others’ approval	1 (StAR [8,19])
		Prompt intention formation	1 (Text4Heart [21,29])
		Prompt review of behavioral goals	1 (Text4Heart [21,29])
		Provide feedback on performance	1 (Text4Heart [21,29])
		Teach to use prompts or cues	1 (TEXT ME [9,19,32])
		Provide opportunities for social comparison	1 (Text4Heart [21,29])
		Plan social support or social change	1 (Text4Heart [21,29])
		Relapse prevention	1 (TEXT ME [9,19,32])
		Stress management	1 (Text4Heart [21,29])
**Intervention development**			
	**Message content based on**		
		Clinical guidelines	5 (TEXT ME [9,19,32], Text4Heart [21,29], Islam [22,33], Heart [24-26,30], and StAR [8,18])
		Expert opinion	2 (Islam [22,33] and StAR [8,18])
		Qualitative patient interviews	1 (Heart [24-26,30])
	**Other intervention characteristics (timing, frequency, directionality, etc) based on**		
		Literature reviews	3 (TEXT ME [9,19,32], Text4Heart [21,29], and StAR [8,18])
		Primary research	2 (Heart [24-26,30] and StAR [8,18])
	**End-user feedback gathered**		
		Yes (questionnaires)	4 (Heart [24-26,30], Text4Heart [21,29], TEXT ME [9,19,32], and StAR [8,18])
		Yes (semistructured interviews)	2 (Text4Heart [21,29] and Islam [22,33])
		Yes (focus groups)	1 (StAR [8,18])
		No	0
**Intervention pretesting**			
		Yes	5 (TEXT ME [9,19,32], Text4Heart [21,29], Islam [22,33], Heart [24-26,30], and StAR [8,18])
		No	0

^a^TEXT ME: Tobacco, Exercise and Diet Messages.

^b^Text4Heart: Text message and Internet-based comprehensive cardiac rehabilitation intervention.

^c^Heart: Heart Exercise And Remote Technologies.

^d^StAR: Mobile Phone Text Messages to Support Treatment Adherence in Adults With High Blood Pressure.

#### Intervention Development

A number of different sources were used to inform the content of the messages, namely, clinical guidelines (all interventions), expert opinion (Islam [22,33], StAR [8,18], and (Heart [24-26,30]), and feedback from qualitative formative work (Heart [24-26,30]; see Multimedia Appendix 4). A number of different professionals were involved in the development of the messages, including clinicians (all interventions), academics (TEXT ME [9,19,32], Islam [22,33], Heart [24-26,30], and StAR [8,18]), health psychologists (Text4Heart [21,29]), exercise physiologists and cardiologists (Heart [24-26,30]), and patient and public representatives (TEXT ME [9,19,32], StAR [8,18], and Text4Heart [21,29]). The frequency and timing of the messages, intervention duration, directionality, and level of tailoring were determined based on literature reviews (TEXT ME [9,19,32], StAR [8,18], and Text4Heart [21,29]), qualitative research (Heart [24-26,30] and StAR [8,18]), and previous similar studies undertaken by the authors (Text4Heart [21,29] and Heart [24-26,30]).

End-user feedback was gathered to inform the development of all the interventions (Table 3). Feedback was most frequently gathered through structured questionnaires (TEXT ME [9,19,32], Text4Heart [21,29], Islam [22,33], and Heart [24-26,30]). Some studies also used qualitative methods, namely, semistructured interviews (Text4Heart [21,29] and StAR [8,18]) and focus groups (StAR [8,18]). Readability, acceptability, and perceived utility were the aspects more frequently examined. End users’ feedback contributed to the refinement of all the interventions (simplifying the content of the messages, changing their tone, and increasing the level of tailoring, among others).

#### Intervention Pretesting

All the interventions were pretested before being formally evaluated in a clinical trial. Pretesting activities involved pilot studies with a purposive or convenience sample of 10 to 30 participants who received the text messages for a period ranging from 1 to 4 weeks (Table 4). A number of modifications were introduced in all interventions as a result of the pilot studies. They included the refinement of the message bank and software system to deliver them (TEXT ME [9,19,32]), increasing the level of tailoring and modifying the website system (Text4Heart [21,29]), changing the timing of the messages (Islam [22,33] and StAR [8,18]), including all SMS messages on a website for the participants to review retrospectively if they chose (Heart [24-26,30]), and allowing the participants to change the language of the messages (StAR [8,18]).

**Table 2:** Intervention design: use of theory and behavior change techniques.

Trial	Theoretical approach adopted for intervention	In what way were theories used to develop the intervention	Behavior change techniques used
TEXT ME^a^ [9,19,32]	Control theory, information-motivation-behavioral skills model, operant conditioning, social-cognitive theory, theory of planned behavior, and theory of reasoned action	Select intervention techniques	Provide information about behavior-health link, provide information on consequences, prompt barrier identification, provide general encouragement, set graded tasks, provide instruction, prompt self-monitoring of behavior, teach to use prompts or cues, relapse prevention, and time management
Text4Heart^b^ [21,29]	Social-cognitive theory and common sense model	Select and develop intervention techniques	Provide information about behavior-health link, provide information on consequences, prompt intention formation, prompt barrier identification, provide general encouragement, set graded tasks, provide instruction, model or demonstrate the behavior, prompt specific goal setting, prompt review of behavioral goals, prompt self-monitoring of behavior, provide feedback on performance, provide opportunities for social comparison, plan social support or social change, stress management, time management, and interpretation and normalizing of physical or emotional symptoms when changing behavior
Islam [22,33]	Behavioral learning theory and transtheoretical model of behavioral change	Select and develop intervention techniques	Reinforce and encourage healthy behavior and lifestyle modification, stimuli for medication adherence—using behavior learning techniques—and inform, motivate, and provide psychological support—using transtheoretical model of behavior change techniques
Heart^c^ [24-26,30]	Self-efficacy theory framework	Select and develop intervention techniques	Prompt barrier identification; provide general encouragement; model or demonstrate the behavior; prompt specific goal setting; time management; coping efficacy, self-regulation, social support; scheduling efficacy; interpreting physiology, including somatic and emotional states; and exercise prescription
StAR^d^ [8,18]	Integrated theory of behavior change	Select intervention techniques and tailor intervention techniques to recipients	Provide information about behavior-health link, provide information on consequences, provide information about others’ approval, provide general encouragement, and prompt specific goal setting

^a^TEXT ME: Tobacco, Exercise and Diet Messages.

^b^Text4Heart: Text message and Internet-based comprehensive cardiac rehabilitation intervention.

^c^Heart: Heart Exercise And Remote Technologies.

^d^StAR: Mobile Phone Text Messages to Support Treatment Adherence in Adults With High Blood Pressure.

#### Interventions With Development Details Not Available

Additional information about intervention development was not available from authors for 4 of the 9 trials identified in the Text2PreventCVD systematic review [16,17,23,31,34]. Only 1 of them replied to our invitations, but they were unable to share data in the time specified for the analysis. These interventions were heterogeneous in scope (targeting diabetes prevention [31] and control [16,17], hypertension [23], and adherence to cardiovascular preventive treatment [34]) and setting (inner city emergency department in the United States [16,17], ambulatory care in Russia [23], community of working men in India [31], and primary care general practices in England [34]). The intervention planning was only reported in 1 of the interventions (Text-Med intervention [16,17], which used qualitative research methods to elicit user views of the planned behavior changes. The process of designing the intervention was reported only in 1 of the studies [31], which used the transtheoretical model of behavioral change. Intervention development and pretesting was only reported by 1 intervention (Text-Med intervention [16,17], which used clinical guidelines, expert opinions, and qualitative research with patients to develop the intervention, pretesting it in a 1-week pilot trial with 23 participants). Additional details about the development process of the rest of the interventions were not identified in the available publications.

**Table 3:** Intervention development: end-user feedback.

Trial	Methods used to gather end-user feedback	Aspects intended to be examined through end-user feedback	Changes in the intervention implemented as a result of end users’ feedback
TEXT ME^a^ [9,19,32]	Data collection: questionnaires (n: 53); sampling: opportunistic	Readability and perceived utility	Stop sending messages about eating meat products to vegetarians and grammatical suggestions
Text4Heart^b^ [21,29]	Data collection: website usage statistics (n=85), mobile phone usage survey (n=74), intervention feedback surveys (n=85), and intervention feedback semistructured interviews (n=17); sampling: opportunistic	Choice of technology to deliver the messages, acceptability of physical activity messages, level of tailoring, and directionality of messages	Intervention delivered by short message service only (too few end users had smartphones at the time), changes made to content of exercise prescription messages, higher level of tailoring (messages personalized with name, time of day to receive messages, and primary behavior targeted), and 2-way messaging (participants could text in questions and receive a personal reply)
Islam [22,33]	Data collection: face-to-face interviews using structured questionnaires (n=50); sampling: purposive	Readability, acceptability, and perceived	Messages made simpler (only 1 content per message)
Heart^c^ [24-26,30]	Data collection: Web-based survey (n=20); sampling: purposive	Readability, acceptability, perceived utility, and persuasiveness	Actions taken to resolve technical difficulties encountered by participants (eg, written instructions about how to log on to the study website)
StAR^d^ [8,18]	Data collection: semistructured interviews and focus groups with patients (n=35), primary care providers (n=12), health systems managers (n=5), and chronic dispensing service providers (n=3); sampling: purposive	Readability, acceptability, perceived utility, and persuasiveness	Tone, use of abbreviations, and addition of named provider to sign off message

^a^TEXT ME: Tobacco, Exercise and Diet Messages.

^b^Text4Heart: Text message and Internet-based comprehensive cardiac rehabilitation intervention.

^c^Heart: Heart Exercise And Remote Technologies.

^d^StAR: Mobile Phone Text Messages to Support Treatment Adherence in Adults With High Blood Pressure.

**Table 4:** Intervention pretesting.

Trial	Pretesting methods	Changes in the intervention implemented as a result of the pretesting exercise
TEXT ME^a^ [9,19,32]	n=16; sampling: convenient; additional details: The aim of the pilot testing was to evaluate the messages and the system to deliver them. Participants received the relevant messages for 1 week. At the conclusion of the pilot study, participants received a questionnaire asking for the potential impact of the messages to change behavior, the process of receiving messages (eg, timing, personalization, and frequency), and for any further feedback	Refinement of the message bank and software system
Text4Heart^b^ [21,29]	n=20; sampling: convenient; additional details: The aim of the pilot testing was to test the healthy eating messages. Participants received 1 text message per day (28 in total) and had access to the supporting website; 4 weeks later, participants were contacted by text and email to complete a follow-up Web-based survey. Website usage statistics including the frequency, login period, and page views were also tracked	No changes made to healthy eating message content or tone (viewed as acceptable), self-efficacy included as underlying theoretical construct; higher level of tailoring (messages personalized with name, time of day to receive messages, and primary behavior targeted), website log-on system simplified
Islam [22,33]	n=30; sampling: purposive; additional details: intervention pretested in patients with type 2 diabetes selected from a diabetes clinic of a tertiary hospital	Timing of text messages was set to be delivered from 10 am to 5 pm
Heart^c^ [24-26,30]	n=10; sampling: convenience; additional details: Pilot testing of the intervention was integrated into the full randomized controlled trial (during the study, the first 10 study participants were closely monitored for 6 weeks to ensure that they received the messages and to resolve technical issues)	All SMS^d^ messages were made available on a website for participants to review retrospectively if they chose and correction of grammatical errors identified by participants
StAR^e^ [8,18]	n=19; sampling: purposive; additional details: The full intervention package was tested in the 3 languages most commonly used in Cape Town (English, Afrikaans, and isiXhosa). Participants were contacted on a weekly basis by a researcher for a semi-structured interview on their experience of the intervention and the SMS text message delivery system	Change timing of the message and allowing users to change language

^a^TEXT ME: Tobacco, Exercise and Diet Messages.

^b^Text4Heart: Text message and Internet-based comprehensive cardiac rehabilitation intervention.

^c^Heart: Heart Exercise And Remote Technologies.

^d^SMS: short message service.

^e^StAR: Mobile Phone Text Messages to Support Treatment Adherence in Adults With High Blood Pressure.

## Discussion

### Summary of Findings

In this study, we conducted an in-depth examination of the development process for 5 of the 9 mHealth interventions identified as a part of the Text2PreventCVD meta-analysis. We observed that the development process of these interventions was complex and comprehensive, involving formative work with multiple studies using both quantitative and qualitative designs to gather evidence to inform decisions about the scope, content, and structure of the intended interventions. Although formative studies varied in terms of the way the interventions were developed, the following processes were identified in all the interventions: use of theory and behavior change techniques for intervention design, use of end-user feedback for intervention development, and pilot studies for intervention pretesting.

### Comparison of Findings With Previous Literature

It has been argued that, as in any health promotion practice, quality, rigor, and careful development of mHealth interventions remain essential [49]. However, a number of recent systematic reviews highlight that a significant proportion of health behavior interventions are not based on theory [50-52], do not follow a user-centered approach [11,53], and lack adequate pretesting procedures [54,55]. Indeed, it has been argued that the SMS text message development is commonly hidden within a black box, lacking deconstruction of the steps involved in creating messages as well as rigorous evaluations of the quality of the health communication messages [14]. None of this is supported by findings from our study, which showed that the development process of the interventions contributing to the Text2PreventCVD meta-analysis included those elements. At least 3 factors could contribute to explaining this apparent discrepancy. First, as opposed to previous reviews, our findings are mostly based on data supplied by the authors of the studies themselves rather than merely by information in published manuscripts. Therefore, it is plausible that some interventions were not underdeveloped but rather underreported. Previous studies have already noted that the documentation and critical analysis of the formative research process required in the development and refinement of messages is a neglected area in the published literature [56]. For quality assessment purposes and to improve future research and program implementation, this research should be accessible for scientific scrutiny. This should include a description of the development of the SMS content (and quality of the evidence on which the messages are based), the theoretical basis of the messages (and the rationale for choosing it), cultural adaptation to the target population, and context along with an analysis of how the content is received by users [54,57,58]. This would enable translation and assessment of generalizability of findings of the research. Second, although all the examined interventions included as part of their development the use of theory, user feedback, and pretesting activities, it could be argued that these elements were not rigorously applied, a common criticism in the current literature [11,37,52]. This may be particularly relevant with the use of theory; superficial use of theory in the development of health behavior interventions has been identified as one of the main barriers hindering progress in quantifying its impact on behavior change [52]. Third, almost half of the interventions initially identified were not able to be included, and this could have resulted in a selection bias (leading to an overrepresentation of more comprehensively developed interventions), as discussed as part of the limitations below.

### Implications for Future Research

Future studies are needed to explore the extent to which the development process is associated with intervention success and to identify *active* *ingredients* in the development process. To do so, valid and reliable measures of intervention development complexity are very much needed as well as data from a larger number of more heterogeneously developed interventions. Publication bias (a common source of potential bias in systematic reviews [59]) should also be adequately accounted for in such studies, as less successful interventions may be less likely to be published and may be less likely to have followed a thorough development process. Intervention success should not only be evaluated in terms of clinical effectiveness observed within the context of a clinical trial but also in terms of research translation capacity, that is, the extent to which the interventions are successfully implemented at the population level and are acceptable and perceived as useful by the target populations.

### Strengths and Weaknesses

This study provides for the first time a detailed and systematic description of the development process of mHealth interventions to support behavior change in people with CVD. The data described in our study were initially extracted from available publications and subsequently reviewed by the authors of the relevant trials to maximize their accuracy and completeness. Our study has some limitations. First, although 9 trials were identified in the Text2PreventCVD meta-analysis, there was no contribution beyond published data for 4 of them (ie, the information we initially extracted from the publications was not reviewed and added by the developers). This could bias our results if the reason for not providing further data was related to the methods used to develop those interventions. Second, in our study, we could not examine the important question of whether more comprehensive development processes lead to more successful interventions. The main reason for not being able to explore this was the lack of sufficient variability across intervention development methods. As shown in Table 1, all the interventions included in this study were developed using complex and comprehensive methods: all of them carried out studies to identify the key behavioral issues, needs, and challenges that the planned intervention was intended to address; all of them used 1 or more theory and behavior change techniques to design the intervention; all developed the messages based on a number of different information sources; all gathered end-user feedback; and all conducted feasibility studies to pretest the interventions.

### Conclusions

A detailed examination of the development of SMS interventions to improve CVD showed the processes used were complex and comprehensive. Our study identified a wide range of different methodologies to inform intervention planning, intervention design, intervention development, and intervention pretesting. Literature review and stakeholder consultations were the most frequently used methods to guide intervention planning and design. Regarding the development of the interventions, clinical guidelines and expert consultations were the main information sources for message content, whereas literature reviews and end-user feedback were the main source to inform decisions about other components of the interventions such as timing, directionality, or level of tailoring. Pilot studies with relatively small sample sizes were frequently used for intervention pretesting. Further work is needed to establish whether effective messaging systems can be adapted from work already done or whether this level of development is always needed for application in other conditions and settings. Additional research is also needed to examine the potential association between development methods and intervention success as well as to identify *active* *ingredients* in the development methods.

## Appendix

Multimedia Appendix 1 Characteristics of the interventions (message customization, personalization, delivery timing, frequency, message content, directionality, character set).

Multimedia Appendix 2 Platform characteristics.

Multimedia Appendix 3 Intervention planning: information sources used to identify the key behavioral issues, needs, and challenges that the planned intervention was intended to address.

Multimedia Appendix 4 Intervention development: sources used to inform the development of the intervention.

## Abbreviations

CVD: cardiovascular disease
Heart: Heart Exercise And Remote Technologies
mHealth: mobile health
NIHR: National Institute for Health Research
SMS: short message service
StAR: Mobile Phone Text Messages to Support Treatment Adherence in Adults With High Blood Pressure
Text4Heart: Text message and Internet-based comprehensive cardiac rehabilitation intervention
TEXT ME: Tobacco, Exercise and Diet Messages
