# Supplementary material for: Examining Development Processes for Text Messaging Interventions to Prevent Cardiovascular Disease: Systematic Literature Review
Source: JMIR Mhealth Uhealth. 2019 Mar 29;7(3):e12191. doi: 10.2196/12191 (PMC6460311; doi:10.2196/12191)
Supplement: Multimedia Appendix 1 [file mhealth_v7i3e12191_app1.pdf]

Multimedia Appendix 1a. Characteristics of the interventions (message customization, personalization, delivery timing, and frequency)

**Trial/ Country (refs)**	**Message customization**			**Personalization**	**Delivery timing**			**Frequency**	
	Generic v individualized	Ability to update custom settings	Simple v complex customization		Timing	Random times	Specific times	Number of messages per day or per week	Fixed v variable frequency
TEXT ME/ Australia [9, 19, 32]	Content was partly generic and partly customized to participant's needs.	Customization was only at baseline	Relatively simple algorithms using minimal baseline data	Yes	On working days during working hours. Occasional season's greetings message on holidays	Random delivery times on random weekdays	No (network latency times cannot be measured with confidence).	1 message per day, average 4 messages per week	Consistent schedule of 4 messages per week on random weekdays.
Text4Heart/ New Zealand [21, 29]	Individualized timing	Custom settings could be updated but require participants to phone research centre to request a change	Simple	Yes- messages tailored to participants' name	Preferred time of day to receive messages	No	Yes	7 messages per week (1 per day). From weeks 13 to 24, the frequency of messages decreased to 5 per week.	Variable: 7 messages per week from weeks 1 – 12; 5 messages per week from weeks 13-24
Islam/ Bangladesh [22, 33]	Generic	Message delivery software allowed to update custom settings	Not applicable	Not personalized	Random	Between 10 am-5 pm	None	One message per day during a 6 months period	Fixed
Heart/ New Zealand [24-26, 30]	Individualized depending on prescribed exercise for each<br><br>Week, including duration, frequency, and intensity of exercise	Participants could change their ex prescription but had to phone in to research centre to do so	Simple	Personalised to baseline fitness level obtained in Peak VO2 test	Random	Sent randomly during daytime hours		118 messages over 24 weeks	Six messages per week for the first 12 weeks, five messages per week for 6 weeks and then four messages per week for the remaining 6 weeks

StAR/ South Africa [8, 18]	Messages were made available in participants' preferred language (English, isiXhosa and Afrikaans) and timing of the messages.	Customise at baseline, offered option to change language, timing after enrolment using automated system	Simple	Yes, some messages included the name of patients' own clinic or pharmacy or health care worker	Timing according to participants' preferences	No	Yes, timing of some message was related to clinic appointments and medicine collection (information facilitated by secure linkage to computerized appointment data)	Information-only group: 1 message sent weekly for 12-months.<br><br>Interactive SMS group: SMS-text message sent weekly for 12-months.	Fixed

Multimedia Appendix 1b. Characteristics of the interventions (message content, directionality, character set)

Trial/ Country (refs)	Message content					Directionality	Character set
	**Sender signature included in the messages**	**Readability level**	**Message length**	**Order of message content (random v sequential)**	**Unique messaging v repetition**	**Unidirectional v bidirectional**	**Unicode allowed?**
TEXT ME/ Australia [9, 19, 32]	A full signature was included in the first message. Subsequent messages used abbreviated signature.	5th-8th - grade reading level	120-160	Random order.	Unique	One-way	Capacity to support Unicode (but not implemented).
Text4Heart/ New Zealand [21, 29]	Yes, each message began with T4H (Text4Heart)	Appropriate reading level (rms 800 lexile: approximately age 13 years)	140 characters or less	Sequential: general informational messages regarding basic heart health sent first, followed by specific behaviour change strategies	Unique (except weekly pedometer count reminder)	Two-way interaction: bidirectional messaging was used because participants were prompted to text in their weekly pedometer step counts and to ask questions or for feedback on other behaviours.	Unclear
Islam/ Bangladesh [22, 33]	Yes	Readability level not tested, but written using language suitable for those who completed primary education	160 characters or less	Random order	Repetition (all the messages developed were sent twice during the 6 months period)	One-way	Not applicable
Heart/ New Zealand [24-26, 30]	Yes, each message began with HEART:	Readability level not tested, but written in plain language, and pre-tested with end-users	140 characters or less	Sequential	Unique	One way	Unicode not allowed
StAR/ South Africa [8, 18]	Yes	5th-8th - grade reading level	160 characters or less	Random	Unique	Two intervention arms were included: one with information only SMS - texts, and one that included an interactive component.	Yes (used @ for example)
