# Supplementary material for: Examining Development Processes for Text Messaging Interventions to Prevent Cardiovascular Disease: Systematic Literature Review
Source: JMIR Mhealth Uhealth. 2019 Mar 29;7(3):e12191. doi: 10.2196/12191 (PMC6460311; doi:10.2196/12191)
Supplement: Multimedia Appendix 2 [file mhealth_v7i3e12191_app2.pdf]

Multimedia Appendix 2. Platform characteristics

Trial/ Country (refs)	Platform			Payment model
	**Name of the platform**	**Who set the platforms up**	**Who administered the platform**	
TEXT ME/ Australia [9, 19, 32]	Custom programmed platform that worked through an API interface with a commercial gateway company for sending messages	Programmers	Engineering member of the team.	Free to trial participants
Text4Heart/ New Zealand [21, 29]	MessageMedia	NIHI's IT staff set up the content delivery system and monitored the system	NIHI's IT staff set up the content delivery system and monitored the system	Each intervention participant received a nz$20 telephone credit voucher to reimburse any costs associated with replying to intervention messages.
Islam/ Bangladesh [22, 33]	Web-based message delivery manager software provided by Grameenphone Bangladesh	Grameenphone Bangladesh provided the software which was customized by the Principal Investigator.	Study Investigators	Bulk-bill at the end of each month to the research institute. Cost per message 0.0125 USD. Messages were free for the participants.
Heart/ New Zealand [24-26, 30]	HEART messaging platform	A bespoke messaging platform was developed by IT team at the National Institute for Health Innovation (NIHI). The platform contained the message library and decision tree for sending messages via the gateway company	NIHI's IT team	Research funding paid for salary cost for developers of the platform and to maintain the system.<br><br>A gateway company was hired to deliver the messages. Messages were zero-rated so participants were not charged for messages
StAR/ South Africa [8, 18]	All SMS text messages were delivered automatically via an open source Web-based electronic medical record system (OpenMRS version 1.6.1, OpenMRS Ltd, Grandville, MI).	Programmers	Study Investigators	Free to user
