# Supplementary material for: Examining Development Processes for Text Messaging Interventions to Prevent Cardiovascular Disease: Systematic Literature Review
Source: JMIR Mhealth Uhealth. 2019 Mar 29;7(3):e12191. doi: 10.2196/12191 (PMC6460311; doi:10.2196/12191)
Supplement: Multimedia Appendix 3 [file mhealth_v7i3e12191_app3.pdf]

Multimedia Appendix 3. Intervention planning: information sources used to identify the key behavioral issues, needs, and challenges that the planned intervention was intended to address

Trial/ Country (refs)	Information sources
TEXT ME/ Australia [9, 19, 32]	• Literature review<br>• Examination of relevant behavior change theories<br>• Stakeholder consultation with key peak organizations<br>• Consumer surveys
Text4Heart/ New Zealand [21, 29]	• Systematic literature review<br>• Examination of relevant theory and behavior change techniques<br>• Stakeholder consultation with cardiac rehabilitation nurses and cardiologists<br>• Observation of a centre-based cardiac rehabilitation program<br>• Examination of current cardiac rehabilitation guidelines
Islam/ Bangladesh [22, 33]	• Literature review<br>• Consultation with experts and other stakeholders<br>• Group-meetings
Heart/ New Zealand [24-26, 30]	• Primary qualitative research to elicit user views of the planned behavior changes<br>• Consultation with experts, members of user groups, other stakeholders<br>• Examination of relevant theory
StAR/ South Africa [8, 18]	• Systematic literature review of qualitative studies<br>• Primary qualitative research<br>• Consultation with experts and other stakeholders
