# Supplementary material for: Examining Development Processes for Text Messaging Interventions to Prevent Cardiovascular Disease: Systematic Literature Review
Source: JMIR Mhealth Uhealth. 2019 Mar 29;7(3):e12191. doi: 10.2196/12191 (PMC6460311; doi:10.2196/12191)
Supplement: Multimedia Appendix 4 [file mhealth_v7i3e12191_app4.pdf]

Multimedia Appendix 4. Intervention development: sources used to inform the development of the intervention

| Trial | Message content based on: | Professionals involved in development of the message content | Frequency of the messages based on: | Timing of the messages based on: | Duration of the intervention based on: | Directionality of the messages (1-way v 2-ways) based on: | Level of tailoring based on: |
|---|---|---|---|---|---|---|---|
| TEXT ME [9, 19, 32] | • CG | • Cardiologist<br>• Nurses<br>• Dieticians<br>• Psychologists<br>• Public health and primary care physicians<br>• Patients and public representative | • LR | • LR | • LR | • LR | • LR |
| Text4Heart [21, 29] | • CG | • Health psychologist<br>• Cardiac rehabilitation nurses and cardiologists<br>• End-users | • Previous study[a] | • Previous study[a] | • Previous study[a] | • Previous study[a] | • Previous study[a] |
| Islam [22, 33] | • CG<br>• Expert opinion | • Primary care physicians<br>• Diabetologists<br>• Cardiologists<br>• Epidemiologists<br>• Anthropologists<br>• Nurses<br>• Nutritionists<br>• Research Fellows<br>• Medical students | • LR<br>• EC | • LR<br>• EC | • LR<br>• EC | • EC | None |
| Heart [24-26, 30] | • CG<br>• Primary qualitative research | • Investigators<br>• Cardiologists<br>• Nurse specialists<br>• Maori researchers<br>• Exercise physiologists<br>• Behaviour change and public health researchers | • LR | • Primary qualitative research<br>• LR | • LR | • LR | • LR |

| | | | | | | | |
|---|---|---|---|---|---|---|---|
| StAR[8, 18] | • CG<br>• Expert opinion | • Qualitative researchers<br>• Quantitative researchers<br>• Academics<br>• Clinicians<br>• Patient and public representatives | • LR | • Primary qualitative research | • LR | • LR | • LR |

CG, clinical guidelines; EC, expert consultation; LR, literature review

a: Data obtained from a previous similar study (process evaluation and pilot testing of HEART trial)
